# New Water

**DOI:** 10.3201/eid1309.069999

**Published:** 2007-04

**Authors:** Sharon Chmielarz

All those years–almost a hundred–
the farm had hard water.
Hard orange. Buckets lined in orange.
Sink and tub and toilet, too,
once they got running water.
And now, in less than a lifetime,
just by changing the well's location,
in the same yard, mind you,
the water's soft, clear, delicious to drink.
All those years to shake your head over.
Look how sweet life has become;
you can see it in the couple who live here,
their calmness as they sit at their table,
the beauty as they offer you new water to drink.

